# Endoscopic removal of a date pit masquerading as an appendicolith in a young patient with recurrent right lower quadrant pain

**DOI:** 10.1055/a-2954-0379

**Published:** 2026-10-01

**Authors:** Jue-Rong Feng, Xiao-Shi Feng, Zhao-Hui Liu, Yin-Qing Hu, Beibei Li, Qin-Fang Wu, Da-Yong Sun

**Affiliations:** 1 Department of Gastroenterology and Gastrointestinal Endoscopy Center 499778 Shenzhen University First Affiliated Hospital (Shenzhen Second People's Hospital)ShenzhenGuangdongChina

A 27-year-old man presented with a 2-month history of intermittent right lower quadrant pain, aggravated by meals, without fever or vomiting. He had visited two other hospitals; abdominal contrast-enhanced computed tomography was reported as showing mild appendiceal wall thickening with nodular hyperdensities in the appendiceal lumen, while the surrounding fat planes remained clear—findings interpreted as possible chronic appendicitis. No definitive diagnosis had been established. At our institution, physical examination revealed mild tenderness in the right lower quadrant without rebound or guarding. Given his young age and preference for organ preservation, endoscopic direct appendicitis therapy (EDAT) was performed using a 9-Fr cholangioscope (EyeMax; Micro-Tech, Nanjing, China; Video 1 ). The preoperative diagnosis remained uncertain, as imaging was non-specific and the patient had no clear history of foreign body ingestion. Upon entering the appendiceal lumen, a sharp, elongated, brownish foreign body was identified, with its pointed ends embedded in the mucosa ( Fig. 1 ). Using a 0.05-inch retrieval basket, the foreign body was ensnared at its mid-portion and gently extracted under direct visualization ( Fig. 2 ). Post-extraction irrigation confirmed an intact appendiceal wall without perforation or bleeding. The patient's abdominal pain resolved immediately, and he was discharged the following day. At 3-month follow-up, he remained asymptomatic. The retrieved specimen was grossly identified as a date pit based on its characteristic shape, size, and brownish color ( Fig. 3 ). Foreign body-induced appendicitis is rare, and date pits are particularly hazardous due to their sharp ends and indigestible nature. 1​ Unlike previous reports of foreign bodies (e.g., chicken bones) requiring surgical appendectomy, 2​ or cases of non-absorbable foreign bodies (e.g., endoscopic clips) in which conservative management failed, 3​ our case demonstrates the diagnostic and therapeutic value of EDAT. To our knowledge, this is the first report of a date pit causing recurrent appendicitis successfully managed by EDAT with cholangioscopic visualization, thereby preserving the appendix. 4​ ​5​

**Video 1** Sub-appendiscopic retrieval of an impacted date pit causing recurrent appendicitis using a retrieval basket via endoscopic direct appendicitis therapy. Video URI:


**Fig. 1:**
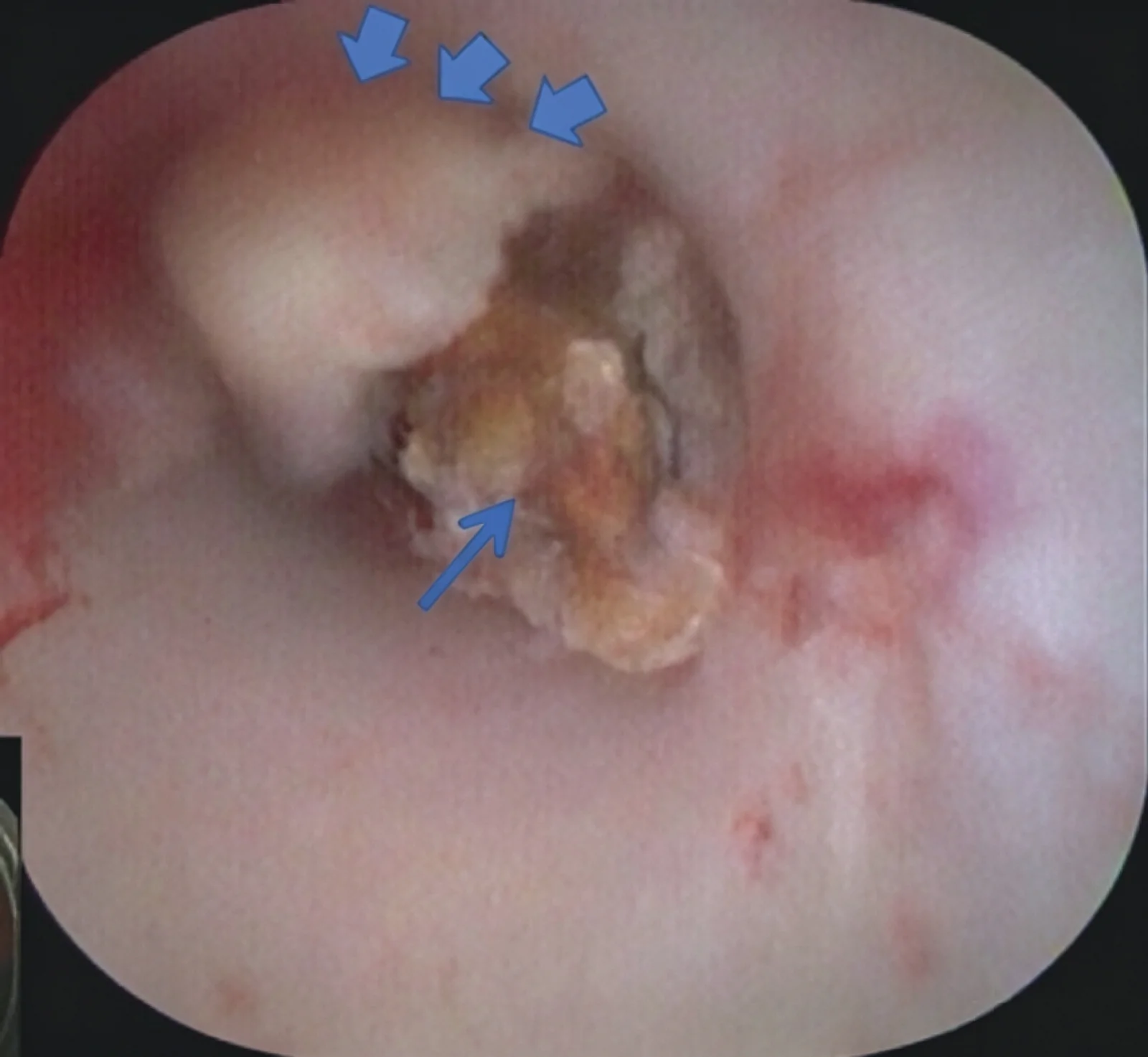
A sub-appendiscopic view showing the impacted date pit (arrow) within the appendiceal lumen, with its pointed ends embedded in the mucosa (arrowheads).

**Fig. 2:**
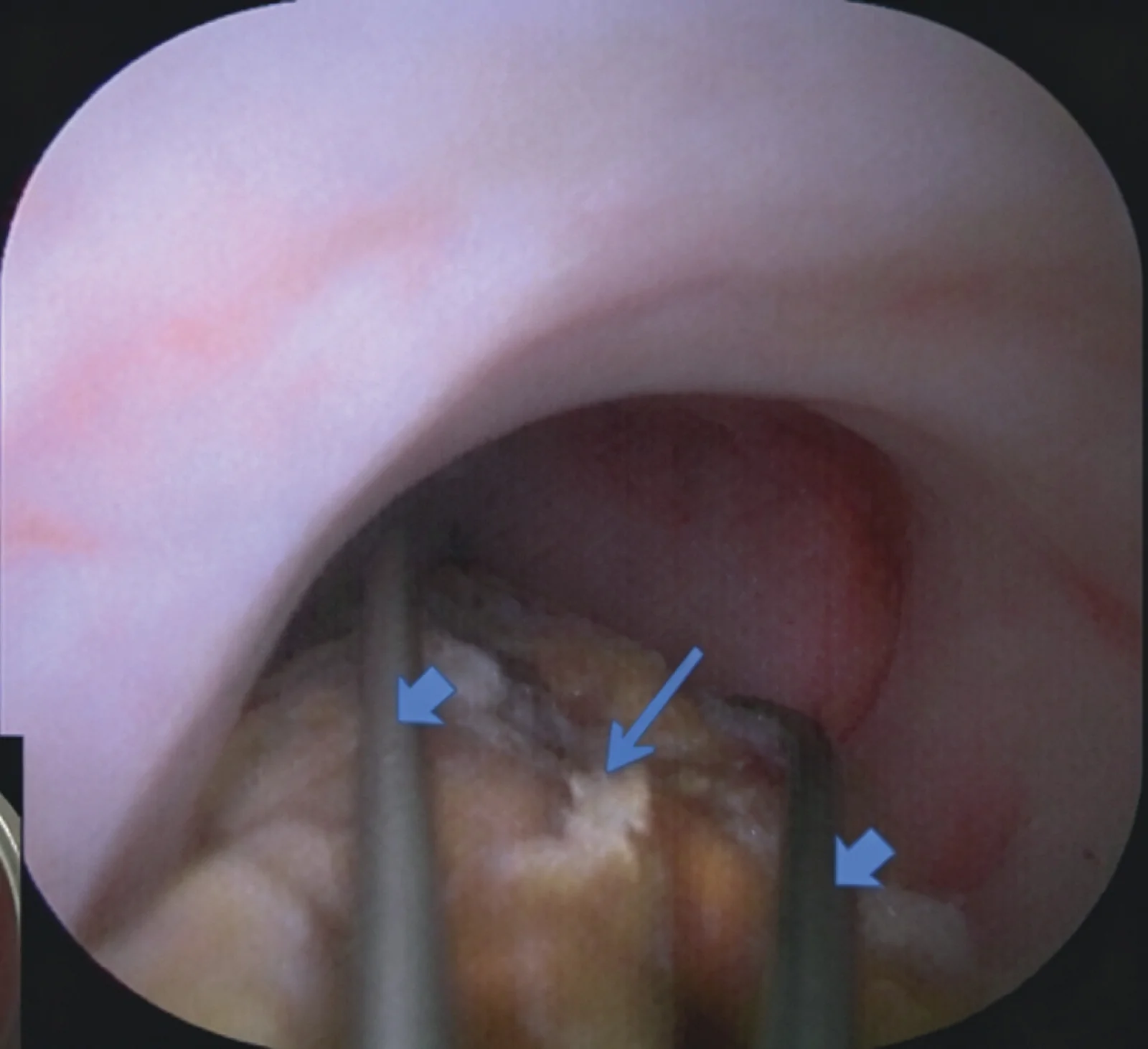
The date pit (arrow) is ensnared at its mid-portion using a 0.05-inch retrieval basket (arrowhead) under direct visualization.

**Fig. 3:**
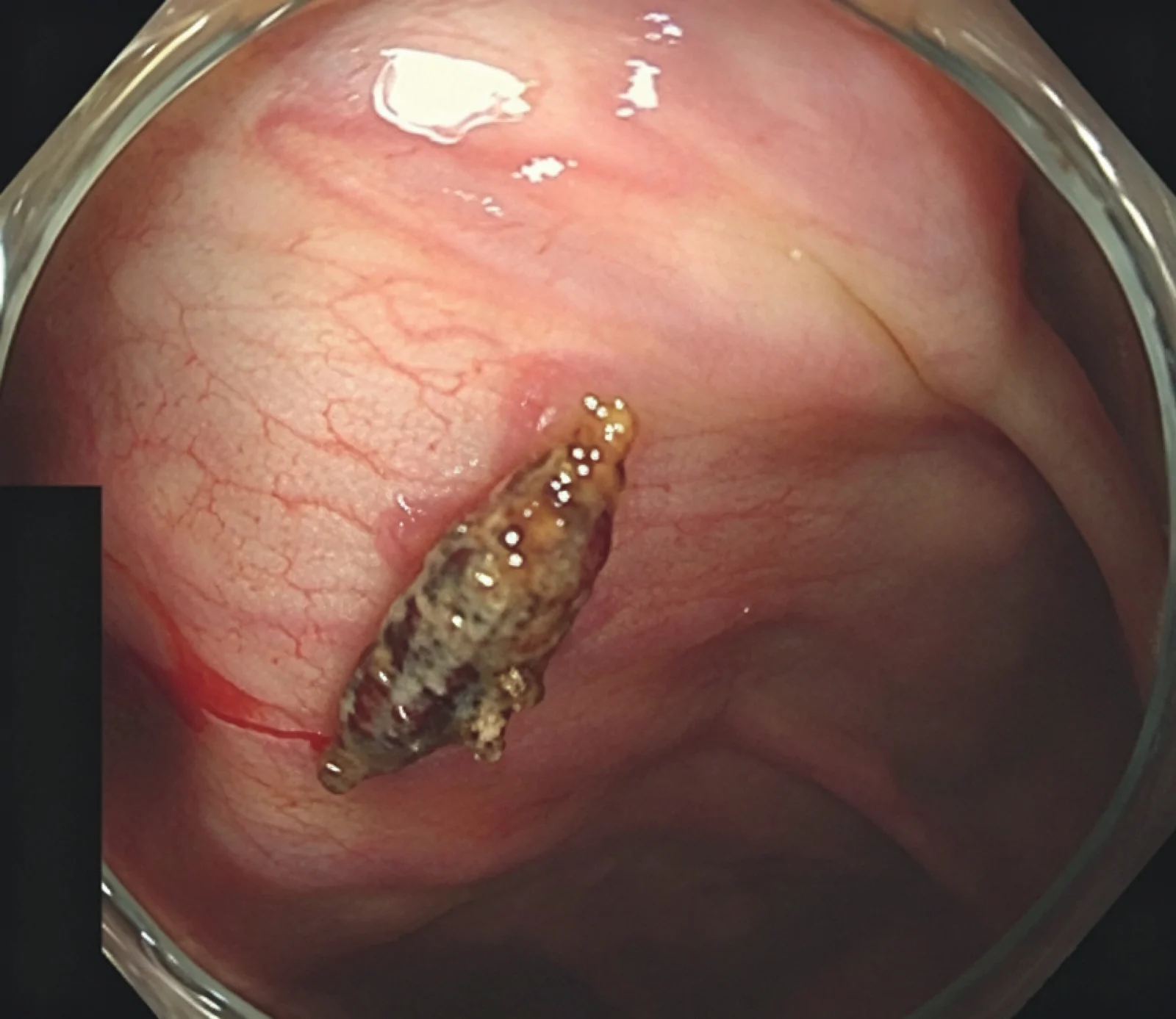
Retrieved specimen grossly identified as a date pit based on its characteristic elongated shape, pointed ends, and brownish color.

Endoscopy_UCTN_Code_TTT_1AQ_2AH

## Declaration of GenAI Use

The authors confirm that they used the DeepSeek artificial intelligence tool to assist with polishing the manuscript.

## References

[1] Klingler P J Seelig M H DeVault K R Ingested foreign bodies within the appendix: a 100-year review of the literature Dig Dis 1999 16 05 308 314 10.1159/000016880 9892790

[2] Huang T Li S K Wang W Chronic abdominal pain caused by foreign bodies in the appendix: a case report World J Gastrointest Surg 2025 17 05 105423 10.4240/wjgs.v17.i5.105423 PMC12149931 40502514

[3] Ono R Yamada T Danbara T Acute appendicitis with periappendiceal abscess caused by migration of an endoscopic clip into the appendix: a case report Cureus 2026 18 03 e105939 10.7759/cureus.105939 PMC13110317 42046642

[4] Cai J Wang Y L Huang S L Acute obstructive appendicitis in a child caused by pinworms treated with endoscopic direct appendicitis therapy Endoscopy 2024 56 E759 E761 10.1055/a-2387-3979 PMC11374444 39231516

[5] Liu B R Song J T Han F Y Endoscopic retrograde appendicitis therapy: a pilot minimally invasive technique in children with acute uncomplicated appendicitis Gastrointest Endosc 2012 76 04 862 866 10.1016/j.gie.2012.05.029 22840292

